# Evaluation test and analysis of a microneedle and iontophoresis based medical device “CELLADEEP Patch” in skin improvement on ex vivo human‐derived skin tissue models

**DOI:** 10.1111/srt.13784

**Published:** 2024-07-19

**Authors:** Xin Rui Zhang, Yong Xun Jin, Pham Ngoc Chien, Trinh Thi Thuy Tien, Shu Yi Zhou, Nguyen Ngan Giang, Linh Thi Thuy Le, Sun Young Nam, Chan Yeong Heo

**Affiliations:** ^1^ Department of Plastic and Reconstructive Surgery College of Medicine Seoul National University Seoul South Korea; ^2^ Department of Plastic and Reconstructive Surgery Seoul National University Bundang Hospital Seongnam South Korea; ^3^ Korean Institute of Nonclinical Study Seongnam South Korea; ^4^ Department of Medical Device Development College of Medicine Seoul National University Seoul South Korea; ^5^ Department of Biomedical Science College of Medicine Seoul National University Seoul South Korea

**Keywords:** CELLADEEP Patch, collagen, iontophoresis, microneedle therapy system, skin improvement

## Abstract

**Background:**

Microneedles are tiny needles, typically ranging from tens to hundreds of micrometers in length, used in various medical procedures and treatments. The tested medical device named “CELLADEEP Patch” a dissolvable microneedle therapy system (MTS), made of hyaluronic acid and collagen. And the iontophoresis technique is also applied in the system. The study aimed to evaluate the effectiveness of the “CELLADEEP Patch” in skin improvement.

**Methods:**

Ex vivo human‐derived skin tissue models were used in this study and they were divided into three different groups, namely, the Untreated Group, the Negative Control Group, and the Test Group respectively. The Untreated Group received no treatment measures, the Negative Control Group was exposed to ultraviolet B radiation (UVB) irradiation, and the Test Group was exposed to UVB irradiation and treated with “CELLADEEP Patch”. Skin moisture content, transdermal water loss, and skin elasticity were evaluated by three clinical devices. Additionally, histological staining and related mRNA expression levels were also analyzed.

**Results:**

The results of skin moisture content, transdermal water loss, and skin elasticity evaluation consistently illustrated that the application of “CELLADEEP Patch” led to remarkable skin improvement. And the analysis of histological staining images also confirmed the effectiveness of the “CELLADEEP Patch”, especially for increasing collagen density. Moreover, the upregulation of Collagen type 1 a (COL1A1) and hyaluronan synthase 3 mRNA expression and the decrease of Matrix metalloproteinase 1 (MMP‐1) and Interleukin‐1 beta (IL‐1β) mRNA expression reflected its wrinkle improvement, moisturizing and anti‐inflammation function.

**Conclusion:**

“CELLADEPP Patch”, the MTS combined with the iontophoresis technique, exhibits its effectiveness in moisturizing, skin elasticity improvement, and anti‐inflammatory function when applied to ex vivo human‐derived skin tissue models in experiments. The study has contributed to the understanding of the “CELLADEPP Patch” and laid the foundation for subsequent animal experiments and clinical trials.

## 1 INTRODUCTION

Microneedles are a type of minimally invasive device. The array consists of a number of micron‐sized needles ranging from 25 to 2000 μm in length.^1^, ^2^, ^3^, ^4^ These needles, produced via microfabrication techniques with diverse materials and geometries, can facilitate drug delivery into the subcutaneous layer by bypassing the stratum corneum barrier. They penetrate the stratum corneum and epidermis to create micropores, allowing drug molecules to permeate passively into the dermal layer.^3^, ^5^, ^6^ Remarkably, each needle is long enough to breach the stratum corneum but short enough to avoid stimulating nerve endings, making microneedle administration painless and user‐friendly compared to traditional invasive injections or oral methods, while offering superior functionality.^7^, ^8^, ^9^, ^10^ Microneedles not only enable drugs to bypass first‐pass metabolism and gastrointestinal degradation but also broaden the range of applicable drug types, regardless of molecular weight or hydrophilicity. There is no confirmed evidence suggesting that microneedles cause or increase the risk of skin infection, nor do they appear to affect normal skin functions.^11^ The Microneedle Therapy System patch tested in the research were 250 μm‐long dissolvable microneedles, made of hyaluronic acid (HA) and collagen. It also contains antioxidants such as vitamins and adenoic acid.

In addition, we also use an iontophoresis machine as an auxiliary means to increase the efficiency of drug delivery and absorption. Iontophoresis is a non‐invasive technique used in medicine to administer medication through the skin using a low‐level electric current.^12^, ^13^, ^14^, ^15^ It can enhance transdermal drug delivery by applying a small electric current to facilitate the movement of charged molecules through the skin. When the electrodes are placed in contact with the skin, a low‐level electric current is applied. This current creates an electric field across the skin. The charged molecules in the drug are influenced by the electric field and migrate toward the oppositely charged electrode.^12^, ^16^, ^17^, ^18^, ^19^, ^20^, ^21^, ^22^ The skin has a natural barrier that prevents many substances from passing through easily. However, the electric current created by iontophoresis can temporarily disrupt this barrier, allowing the charged drug molecules to penetrate the skin more effectively. The delivery of drugs through the skin can be significantly enhanced compared to passive diffusion alone by using iontophoresis.^18^, ^23^, ^24^, ^25^

In this study, we hereby created an ex vivo human‐derived skin tissue model and applied the abovementioned devices to evaluate the its effectiveness in skin improvement (wrinkle, inflammation, whitening, and moisturizing). By investigating its effectiveness and potential benefit, our research contributes to public awareness of skin improvement based on “CELLADEEP Patch”.

## 2 MATERIALS AND METHODS

### 2.1 Materials

**FIGURE 1:**
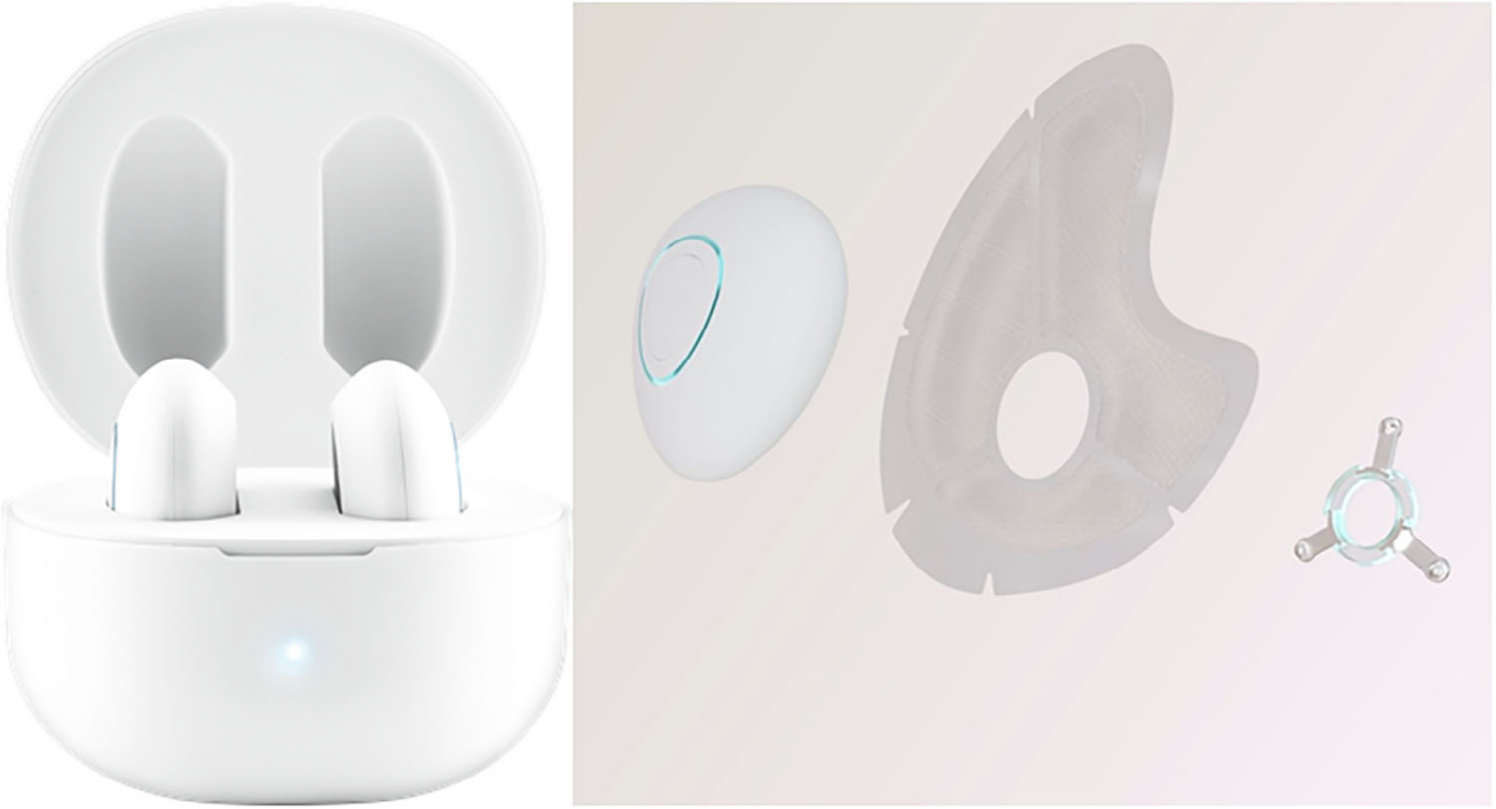
CELLADEEP and CELLADEEP Patch.

CELLADEEP and CELLADEEP Patches were provided by ROOTONIX (Seoul, South Korea) (Figure 1).

### 2.2 Evaluation of skin moisture content

At the time points of before UVB irradiation, 24 h after irradiation, and 48 h after irradiation, skin moisture was measured using Corneometer CM825 (Courage+Khazaka electronic GmbH, Cologne, Germany). The epidermis has the characteristic of high electrical resistance. All electrical phenomena are caused by the movement of charges, and capacitance refers to the ability to store these charges. According to the electric field formed between the anode plates of the probe of this instrument, the capacitance generated at this time can determine the moisture content in the stratum corneum of the epidermis. That is, the measured capacitance is directly proportional to the moisture content in the stratum corneum, so the higher the measured value, the higher the moisture content.

### 2.3 Evaluation of skin water loss

The Tewameter TM300 (Courage+Khazaka electronic GmbH, Cologne, Germany) was used to measure the amount of skin water loss before UVB irradiation, 24 and 48 h after irradiation. This measurement device has an open chamber design on the probe, preventing accumulation of moisture inside, allowing for prolonged measurements. The temperature and humidity sensor inside the probe measures the rate of moisture evaporation over time per unit area of the test site (g/h/m^2^). Utilizing the principle of moisture diffusion, the water loss is calculated based on the increase in relative humidity inside the probe. Additionally, during the initial stage of measurement, the moisture of the skin itself is measured. After approximately 30 to 60 s, the measurement stabilizes, providing accurate values.

### 2.4 Evaluation of skin elasticity

The Cutometer MPA580 (C+K, Germany) was used to measure skin elasticity at time points before UVB irradiation, and 24 and 48 h after irradiation. The analysis focused on parameters R2, R5, and R7 (the closer to 100%, the better the elasticity). This instrument continuously applies suction to the skin three times at a constant pressure of 450 mbar, each time for 2 s, representing skin elasticity through charts and numerical values (Table 1).

**TABLE 1:** Definition and explanation of skin elasticity analysis parameters.

Parameters	Definition and explanation	Summary
R2 (Ua/Uf)	Gross elasticity (resistance to applied physical force, %)	In summary, R2 measures immediate skin deformation, R5 reflects short‐term skin elasticity, and R7 represents long‐term skin elasticity. Higher values of these parameters indicate better skin elasticity, with values closer to 100% indicating optimal elasticity.
R5 (Ur/Ue)	Elastic ratio after the first traction (ratio of inspiratory elasticity to relaxation elasticity, %)	
R7 (Ur/Uf)	Biologic elasticity (ratio of elasticity to total curve, %)	

### 2.5 Hematoxylin and eosin staining

After 48 h of post‐treatment cultivation, the human skin tissue was collected and fixed in 10% formalin, and then embedded in paraffin blocks. Subsequently, tissue sections of 3 μm thickness were made, mounted on slides, and subjected to a rehydration process before staining with hematoxylin and eosin (H&E) solution. Then, the structural and morphological observations of the cross‐sections of the human skin tissue model were performed using an optical microscope (Olympus, Japan).

### 2.6 Masson's trichrome staining

After 48 h of post‐treatment cultivation, the human skin tissue was collected and fixed in 10% formalin, and then embedded in paraffin blocks. Subsequently, tissue sections of 3 μm thickness were made, mounted on slides, and subjected to a rehydration process. Then the slides were soaked in the Bibrich Scarlet‐Acid Fuchsin solution for 5 min, followed by dyeing with phosphotungstic/phosphopolybdic acid for 5 min. Thereafter, collagen synthesis was observed in the cross section of the human‐derived skin tissue model using an optical microscope (Olympus, Japan).

### 2.7 Victoria blue staining

After 48 h of post‐treatment cultivation, the human skin tissue was collected and fixed in 10% formalin, and then embedded in paraffin blocks. Subsequently, tissue sections of 3 μm thickness were made, mounted on slides, and subjected to a rehydration process. After being stained using Potassium Permanganate‐Sulfuric Acid Working Solution, slides were reacted with 1% sodium metabisulfite. Coloration was induced using 70% alcohol, followed by a secondary staining with Nuclear Fast Red stain. Following these procedures, the production level of elastic protein fibers in the cross‐sections of the human skin tissue model was observed using an optical microscope (Olympus, Japan).

### 2.8 Fontana Masson staining

Human‐derived skin tissue was collected at the 48‐h post‐treatment cultivation point of the test product and fixed in 10% formaldehyde to produce paraffin blocks. Subsequently, tissue sections of 3 μm thickness were prepared, mounted on slides, and subjected to hydration process. They were then stained with Ammoniacal Silver Solution for 30 min and rinsed, followed by staining with Gold Chloride Solution 0.2% for 30 s and Sodium Thiosulfate Solution 5% for 1 min, and then washed again. Nuclear Fast Red stain was used for counterstaining. Following these procedures, the degree of melanin and whitening improvement in the cross‐sections of the human‐derived skin tissue model was observed using an optical microscope (Olympus, Japan).

### 2.9 Evaluation of wrinkle and inflammation related cytokines (COL1A1, MMP‐1, and IL‐1β) mRNA expression levels in ex vivo human‐derived skin tissue models

Face skin tissues discarded after surgery from Korean women aged 50 to 70 were used in the study with approval from the KINS Korea Non‐Clinical Research Institute Institutional Review Boards (IRB). Collected human‐derived skin tissues were washed twice with Phosphate Buffer Saline (PBS) and placed in sterilized petri dishes. Then human‐derived skin tissues, cut into 2 cm x 2 cm pieces, excluding the untreated group, were exposed to UVB at an intensity of 200 mJ (BLX‐LMC, VILBER). After UVB exposure, in the test group, a CELLADEEP unit with a bridge and CELLADEEP dedicated patch was attached to the skin and irradiated at the maximum intensity for 1 h. Skin tissues from all groups were punched to a uniform size using an 8 mm Biopsy Punch (KAI Medical), and the underlying adipose tissue was removed as much as possible. A culture medium Dulbecco's Modified Eagle Medium (DMEM), 10% Fetal Bovine Serum (FBS), 1% penicillin/ Streptomycin (P/S) was added to a 6‐well plate, and a Transwell insert (Corning, USA) was attached. Skin tissues were placed on the Transwell insert and cultured for 48 h. After 48 h of culture, skin tissues were homogenized, and Trizol was added. Total RNA was isolated from the skin tissue using the manufacturer's instructions for Total RNA extraction reagent (Takara Bio, Inc.). cDNA synthesis was performed according to the protocol provided by the RevertAid First Strand cDNA Synthesis Kit (Thermo Fisher Scientific, Inc.). For quantitative PCR, TB Green Premix Ex Taq II (Takara Bio, Inc.) and ViiA Real‐Time System (Thermo Fisher Scientific, Inc.) were used with three repetitions. (PCR conditions: denaturation at 95°C for 30 s, 45 cycles of 95°C for 5 s, and 60°C for 34 s. Relative mRNA expression values were determined using the 2^−ΔΔCt^ method.)

### 2.10 Evaluation of moisture related cytokine (HAS3) mRNA expression levels in ex vivo human‐derived skin tissue models

Face skin tissues discarded after surgery from Korean women aged 50 to 70 were used in the study with approval from the KINS Korea Non‐Clinical Research Institute IRB. Collected human‐derived skin tissues were washed twice with PBS and placed in sterilized petri dishes. Then human‐derived skin tissues, cut into 2 cm x 2 cm pieces, were attached to the skin using a CELLADEEP unit with a bridge and CELLADEEP dedicated patch in the test group and irradiated at the maximum intensity for 1 h. Skin tissues from all groups were punched to a uniform size using an 8 mm Biopsy Punch (KAI Medical), and the underlying adipose tissue was removed as much as possible. A culture medium (DMEM, 10% FBS, 1% P/S) was added to a 6‐well plate, and a Transwell insert (Corning, USA) was attached. Skin tissues were placed on the Transwell insert and cultured for 48 h. After 48 h of culture, skin tissues were homogenized, and Trizol was added. Total RNA was isolated from the skin tissue using the manufacturer's instructions for Total RNA extraction reagent (Takara Bio, Inc.). cDNA synthesis was performed according to the protocol provided by the RevertAid First Strand cDNA Synthesis Kit (Thermo Fisher Scientific, Inc.). For quantitative PCR, TB Green Premix Ex Taq II (Takara Bio, Inc.) and ViiA Real‐Time System (Thermo Fisher Scientific, Inc.) were used with 3 repetitions. (PCR conditions: denaturation at 95°C for 30 s, 45 cycles of 95°C for 5 s, and 60°C for 34 s. Relative mRNA expression values were determined using the 2^−ΔΔCt^ method.)

### 2.11 Statistics analysis

All generated data will be statistically analyzed using the SPSS Package Program version 20 (IBM, USA) to determine statistical significance. And the comparison of evaluation results with the control group will be verified using the ANOVA method (^*^ *p *< 0.05, ^**^ *p *< 0.01, ^***^ *p *< 0.001).

The change rate of skin moisture content, water loss, elasticity and mRNA expression were calculated by the following formula.

$$\mathrm{Change}\;\mathrm{Rate}\;\mathrm{of}\;\mathrm{Skin}\;\mathrm{Moisture}\;\mathrm{Content}\left(\%\right)=\frac{\mathrm{Skin}\;\mathrm{Moisture}\;\mathrm{Content}\;\mathrm{of}\;\mathrm{Test}\;\mathrm{Group}-\mathrm{Skin}\;\mathrm{Moisture}\;\mathrm{Content}\;\mathrm{of}\;\mathrm{Negative}\;\mathrm{Control}\;\mathrm{Group}}{\mathrm{Skin}\;\mathrm{Moisture}\;\mathrm{Content}\;\mathrm{of}\;\mathrm{Negative}\;\mathrm{Control}\;\mathrm{Group}}\times 100$$

$$\mathrm{Change}\;\mathrm{Rate}\;\mathrm{of}\;\mathrm{Skin}\;\mathrm{Water}\;\mathrm{Loss}\left(\%\right)=\frac{\mathrm{Skin}\;\mathrm{Water}\;\mathrm{Loss}\;\mathrm{of}\;\mathrm{Test}\;\mathrm{Group}-\mathrm{Skin}\;\mathrm{Water}\;\mathrm{Loss}\;\mathrm{of}\;\mathrm{Negative}\;\mathrm{Control}\;\mathrm{Group}}{\mathrm{Skin}\;\mathrm{Water}\;\mathrm{Loss}\;\mathrm{of}\;\mathrm{Negative}\;\mathrm{Control}\;\mathrm{Group}}\times 100$$

$$\begin{array}{ccc} & & \mathrm{Change}\;\mathrm{Rate}\;\mathrm{of}\;\mathrm{Skin}\;\mathrm{Elasticity}\left(\%\right)\\ & & \;=\frac{\mathrm{Elasticity}\;\mathrm{Parameter}\;\mathrm{Value}\;\mathrm{of}\;\mathrm{Test}\;\mathrm{Group}-\mathrm{Elasticity}\;\mathrm{Parameter}\;\mathrm{Value}\;\mathrm{of}\;\mathrm{Negative}\;\mathrm{Control}\;\mathrm{Group}}{\mathrm{Elasticity}\;\mathrm{Parameter}\;\mathrm{Value}\;\mathrm{of}\;\mathrm{Negative}\;\mathrm{Control}\;\mathrm{Group}}\times 100 \end{array}$$

$$\begin{array}{ccc} & & \mathrm{Change}\;\mathrm{Rate}\;\mathrm{of}\;\mathrm{Wrinkle}\;\mathrm{and}\;\mathrm{Inflammation}\;\mathrm{Related}\;\mathrm{mRNA}\;\mathrm{Expression}\left(\%\right)\\ & & \;=\frac{\mathrm{mRNA}\;\mathrm{Expression}\;\mathrm{of}\;\mathrm{Test}\;\mathrm{Group}-\mathrm{mRNA}\;\mathrm{Expression}\;\mathrm{of}\;\mathrm{Negative}\;\mathrm{Control}\;\mathrm{Group}}{\mathrm{mRNA}\mathrm{Expression}\;\mathrm{of}\;\mathrm{Negative}\;\mathrm{Control}\;\mathrm{Group}}\times 100 \end{array}$$

$$\mathrm{Change}\;\mathrm{Rate}\;\mathrm{of}\;\mathrm{Moisture}\;\mathrm{Related}\;\mathrm{mRNA}\;\mathrm{Expression}\left(\%\right)=\frac{\mathrm{mRNA}\;\mathrm{Expression}\;\mathrm{of}\;\mathrm{Test}\;\mathrm{Group}-\mathrm{mRNA}\;\mathrm{Expression}\;\mathrm{of}\;\mathrm{Untreated}\;\mathrm{Group}}{\mathrm{mRNA}\;\mathrm{Expression}\;\mathrm{of}\;\mathrm{Untreated}\;\mathrm{Group}}\times 100$$

## 3 RESULTS

### 3.1 Evaluation of skin moisture content in ex vivo human‐derived skin tissue models

**FIGURE 2:**
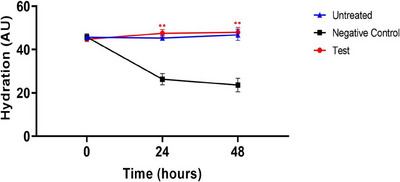
Results of skin moisture content in ex vivo human‐derived skin tissue models (Mean ± error, significance ^*^ *p *< 0.05, ^**^ *p *< 0.01, ^***^ *p *< 0.001).

In ex vivo human‐derived skin tissue models, the skin moisture content was analyzed by using a clinical device named Corneometer. Results showed there was no significant difference in skin moisture levels among all groups at the pre‐UVB exposure time point. However, at the 24‐h post‐UVB exposure time point, the experimental group (CELLADEEP Patch) showed a significant 80.42% increase (improvement) in skin moisture compared to the negative control group (*p* < 0.05). Additionally, at the 48‐h post‐UVB exposure time point, the experimental group (CELLADEEP Patch) exhibited a significant 103.11% increase (improvement) in skin moisture compared to the negative control group (*p *< 0.05) (Figure 2).

### 3.2 Evaluation of skin water loss in ex vivo human‐derived skin tissue models

**FIGURE 3:**
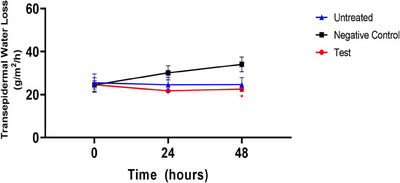
Results of skin water loss in ex vivo human‐derived skin tissue models (Mean ± error, significance ^*^ *p *< 0.05, ^**^ *p *< 0.01, ^***^ *p *< 0.001).

In ex vivo human‐derived skin tissue models, the skin water loss was analyzed by using a clinical device named Tewameter. The pre‐UVB exposure time point did not exhibit any significant variation in skin moisture loss across all groups, according to the results. In contrast to the negative control group, the experimental group (CELLADEEP Patch) showed a trend of 27.75% decrease (improvement) in skin moisture loss at the 24‐h post‐UVB exposure time point. Furthermore, the experimental group (CELLADEEP Patch) showed a noteworthy 33.80% decrease (improvement) in skin moisture loss at the 48‐h post‐UVB exposure time point in comparison to the negative control group (*p *< 0.05) (Figure 3).

### 3.3 Evaluation of skin elasticity in ex vivo human‐derived skin tissue models

#### 3.3.1 R2 (Ua/Uf)

**FIGURE 4:**
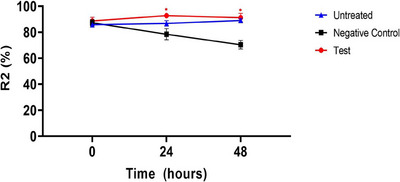
Results of skin elasticity (R2) evaluation in ex vivo human‐derived skin tissue models (Mean ± error, significance ^*^ *p *< 0.05, ^**^ *p *< 0.01, ^***^ *p *< 0.001).

In ex vivo human‐derived skin tissue models, the skin elasticity (R2) was evaluated using a clinical device named Cutometer. As revealed in the following line chart, there was no significant difference in the parameter R2 (%) values (Gross elasticity, resistance to applied physical force, %) for skin elasticity among all groups at the pre‐UVB exposure time point. However, at the 24‐h post‐UVB exposure time point, the test group (CELLADEEP Patch) showed a significant increase (improvement) of 18.18% in R2 (%) values compared to the negative control group (*p* < 0.05). Additionally, at the 48‐h post‐UVB exposure time point, the test group (CELLADEEP Patch) exhibited a significant 29.53% increase (improvement) in R2 (%) values compared to the negative control group (*p *< 0.05) (Figure 4).

#### 3.3.2 R5 (Ur/Ue)

**FIGURE 5:**
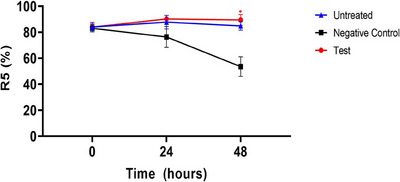
Results of skin elasticity (R5) evaluation in ex vivo human‐derived skin tissue models (Mean ± error, significance ^*^ *p *< 0.05, ^**^ *p *< 0.01, ^***^ *p *< 0.001).

In ex vivo human‐derived skin tissue models, the skin elasticity (R5) was analyzed using a clinical device named Cutometer. Results showed that there was no significant difference between any group at the pre‐UVB exposure time point in the parameter R5 (%) values (ratio of inspiratory elasticity to relaxation elasticity, %) for skin elasticity. In contrast to the negative control group, the test group (CELLADEEP Patch) showed a trend of 18.05% rise (improvement) in R5 (%) values at the 24‐h post‐UVB exposure time point. Additionally, the test group (CELLADEEP Patch) showed a significant 67.01% increase (improvement) in R5 (%) values at the 48‐h post‐UVB exposure time point as compared to the negative control group (*p *< 0.05) (Figure 5).

#### 3.3.3 R7 (Ur/Uf)

**FIGURE 6:**
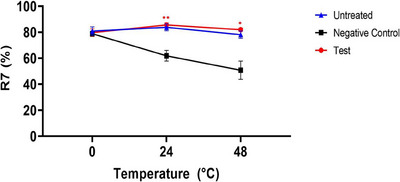
Results of skin elasticity (R7) evaluation in ex vivo human‐derived skin tissue models (Mean ± error, significance ^*^ *p *< 0.05, ^**^ *p *< 0.01, ^***^ *p *< 0.001).

The analysis of skin elasticity (R7) in ex vivo human‐derived skin tissue models using a clinical device named Cutometer showed that there was no significant difference between any group at the pre‐UVB exposure time point in the parameter R7 (%) values (ratio of elasticity to total curve, %) for skin elasticity. In contrast to the negative control group, the test group (CELLADEEP Patch) showed a noteworthy 38.34% rise (improvement) in R7 (%) values at the 24‐h post‐UVB exposure time point. Furthermore, the test group (CELLADEEP Patch) showed a significant 61.35% increase (improvement) in R7 (%) values at the 48‐h post‐UVB exposure time point as compared to the negative control group (*p *< 0.05) (Figure 6).

### 3.4 Histological staining (H&E staining, Masson's trichrome staining, Victoria blue staining, and Fontana Masson staining)

**FIGURE 7:**
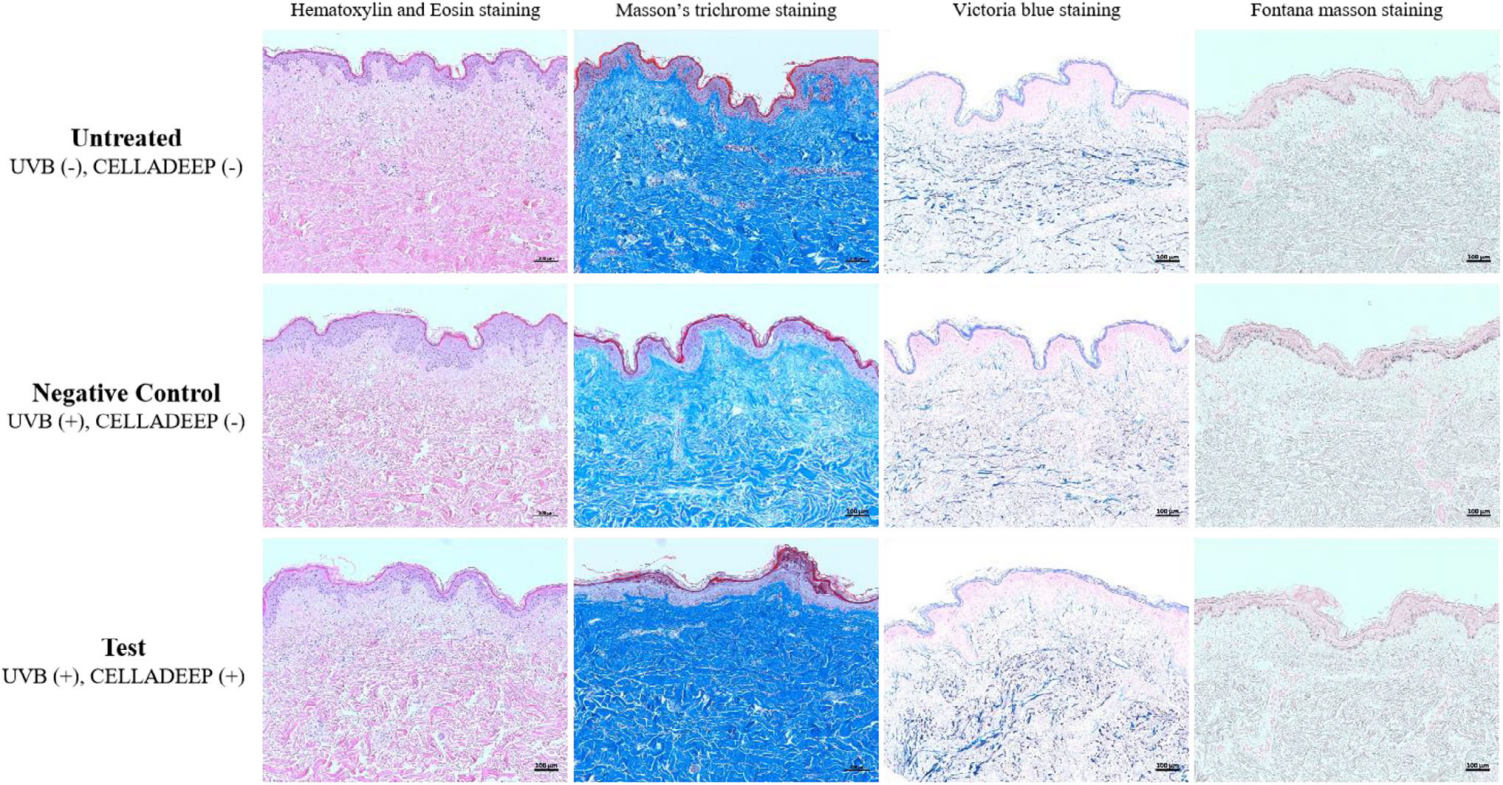
Histological staining images of three different groups (magnification: X100, scale bar: 100 μm).

H&E staining images were taken by an optical microscopy. These images showed that in the negative control group, an increase in epidermal thickness compared to the untreated group was observed due to UVB irradiation. Conversely, in the test group (CELLADEEP Patch), a decrease (improvement) in epidermal thickness compared to the negative control group was observed, with no significant structural or morphological changes noted. In the Masson's trichrome (MT) staining results, a decrease of collagen density in negative control group compared to the untreated group was observed due to UVB irradiation. However, the collagen density in the test group (CELLADEEP Patch) was similar to the untreated group and remarkably higher than the negative control group. The observation of Victoria blue (VB) staining images, elastin in the negative control group was reduced compared to the untreated group by reason of UVB irradiation. Nevertheless, compared to the negative control group, there was an increase (improvement) of elastin in the test group (CELLADEEP Patch). In the Fontana Masson (FM) staining results, it was observed that melanin in the negative control group was higher than the other two groups. And there exist a decrease (improvement) of melanin in the test group (CELLADEEP Patch) (Figure 7).

### 3.5 Evaluation of wrinkle and inflammation related mRNA expression levels in ex vivo human‐derived skin tissue models

**FIGURE 8:**
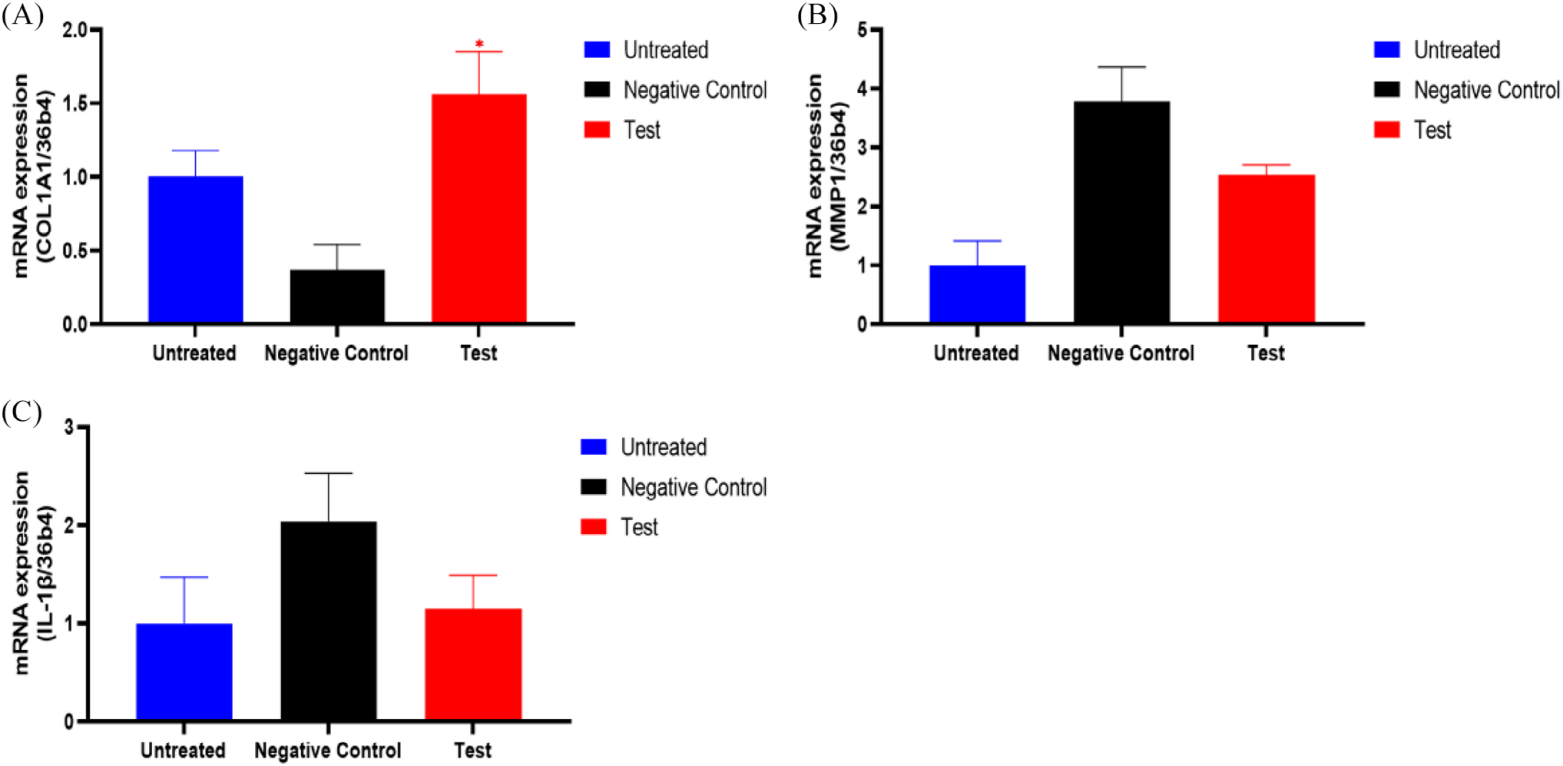
The mRNA expression level of COL1A1, MMP‐1, and IL‐1β.

The analysis based on the mRNA expression of collagen, type I, alpha 1 (COL1A1) revealed a significant 324.31% increase in COL1A1 mRNA expression in the test group (CELLADEEP Patch) compared to the negative control group (*p *< 0.05, Figure 8A). In addition, there was a 33.07% decrease of matrix metalloproteinase‐1 (MMP‐1) mRNA expression in the test group (CELLADEEP Patch) compared to the negative control group (Figure 8B). Similarly, the mRNA expression levels of interleukin 1 beta (IL‐1β) in the test group was 43.88% lower than that in the negative control group (Figure 8C).

### 3.6 Evaluation of moisture related mRNA expression levels in ex vivo human‐derived skin tissue models

**FIGURE 9:**
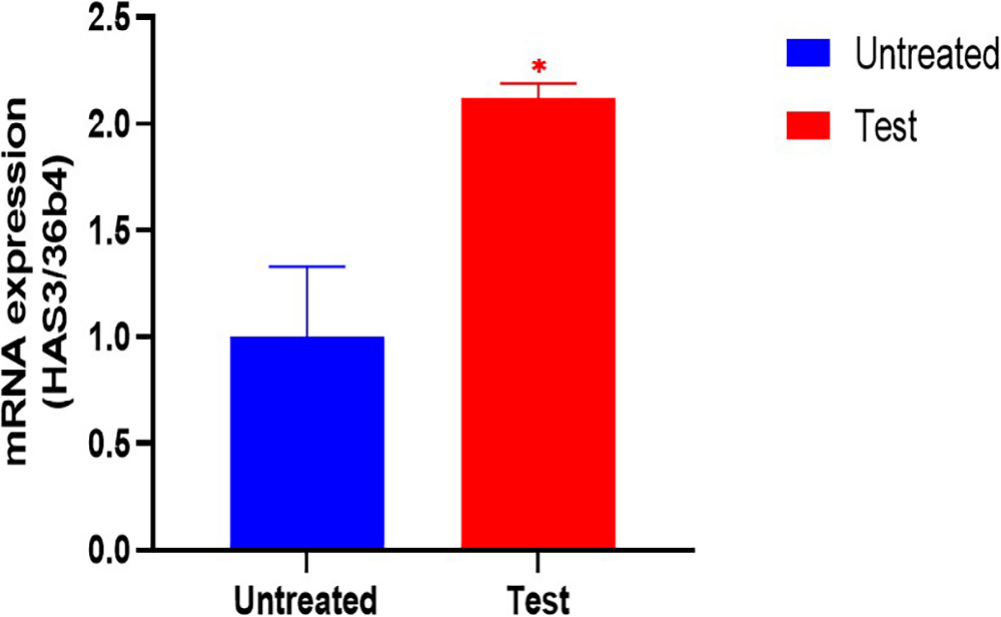
The mRNA expression level of HAS3. HAS3, hyaluronan synthase 3.

In ex vivo human‐derived skin tissue models, the analysis of hyaluronan synthase 3 (HAS3) mRNA expression showed a significant upregulation (improvement) of 111.65% in the test group (CELLADEEP Patch) compared to the untreated group (*p *< 0.05, Figure 9).

## 4 DISCUSSION

Currently, skin care is becoming increasingly important because we live in a society where appearance often plays a crucial role in social interactions,^26^, ^27^, ^28^ having clear, healthy skin can boost self‐confidence and self‐esteem. And societal beauty standards, influenced by media, advertising, and celebrity culture, often prioritize flawless, youthful‐looking skin. In addition, with an aging population, there is growing interest in anti‐aging skincare and treatments. So we developed this kind of medical device for skin improvement.

Hyaluronan, also known as HA, is a naturally occurring substance found in the human body. In the skin, hyaluronan is abundant in the extracellular matrix and plays a crucial role in maintaining skin health.^29^, ^30^, ^31^ One of the primary functions of hyaluronan is to maintain skin hydration. It has a remarkable ability to hold water molecules, helping to keep the skin adequately moisturized. This hydration property gives the skin a plump, supple appearance. In addition, hyaluronan helps in the production of collagen, a protein crucial for skin structure and elasticity. Collagen, combined with hyaluronan, provides firmness and resilience to the skin.^32^ Hyaluronan also acts as a lubricant between the collagen and elastin fibers in the dermis, providing cushioning and protecting the skin from damage caused by friction. Apart from that, hyaluronan participates in the wound‐healing process because it helps regulate inflammation and supports tissue repair by aiding in cell migration and proliferation.^31^, ^33^, ^34^, ^35^, ^36^ HAS3 is an enzyme that in humans is encoded by the HAS3 gene. This protein is involved in the synthesis and production of hyaluronan. It has been proved that HAS3 can influence not only epidermal HA content and thickness but also keratinocyte proliferation and differentiation. The application of the CELLADEEP Patch led to a significant upregulation of HAS3 mRNA expression, indicating that this medical device can benefit skin improvement.

Collagen is a protein that is naturally found in the skin and is responsible for skin strength, elasticity, and youthful appearance. However, as we age, the production of collagen by the body decreases, leading to signs of aging such as wrinkles, sagging skin, and loss of firmness. In skin care, exogenous collagen is often used in the form of topical creams, serums, or supplements to help improve the appearance of the skin.^37^, ^38^, ^39^, ^40^, ^41^ However, it is important to note that the effectiveness of exogenous collagen in skincare products is still a topic of debate among scientists and skincare experts, because collagen molecules are too large to penetrate the skin effectively, and their benefits may be limited. In this study, dissolvable microneedles containing collagen can penetrate the skin without causing significant pain or tissue damage and deliver medications and other therapeutic agents directly into the skin. MT staining images and COL1A1 mRNA expression analysis both reflect the increased amount of collagen in the human‐derived skin tissue model.

Interleukin‐1 beta (IL‐1β) is a pro‐inflammatory cytokine that plays a significant role in the immune response and inflammation, involving in various skin conditions and dermatological disorders. Elevated levels of IL‐1β are often observed in inflammatory skin diseases such as psoriasis and eczema.^42^, ^43^, ^44^ IL‐1β also participates in the development of autoimmune skin diseases. It may contribute to autoimmune skin diseases by promoting inflammation and immune responses against the body's own tissues. For instance, it has been implicated in diseases like systemic lupus erythematosus.^45^, ^46^, ^47^ The assessment of IL‐1β expression indicates that the tested device has good anti‐inflammatory properties, which can help the skin avoid inflammatory reactions.

Moreover, the iontophoresis machine can also enhance transdermal drug delivery by applying a small electric current to facilitate the movement of charged molecules through the skin. And the electric current created by iontophoresis can temporarily disrupt the skin barrier, allowing the charged drug molecules to penetrate the skin more effectively. Research results show that the skin condition has improved in the short term.

## 5 CONCLUSION

In order to evaluate the efficacy of the test product “CELLADEEP Patch” in skin improvement (wrinkles, inflammation, whitening, and moisturizing), application of three clinical devices (Corneometer, Tewameter, Cutometer), histological analysis (H&E staining, MT staining, VB, FM staining), and mRNA expression analysis (COL1A1, MMP‐1, IL‐1ß, HAS‐3) were conducted in ex vivo human‐derived skin tissue models.

For skin moisture content, at the 24‐h and 48‐h post‐UVB exposure time points, the CELLADEEP Patch group showed remarkable increases compared to the negative control group (*p* < 0.05). In addition, there were no significant differences in skin water loss among all groups at the pre‐UVB exposure time point. However, at the 24‐h and 48‐h post‐UVB exposure time points, the CELLADEEP Patch group exhibited significant decreases compared to the negative control group (*p* < 0.05). Moreover, in the analysis of skin elasticity (R2, R5, and R7), no significant differences were observed among all groups at the pre‐UVB exposure time point. However, at the 24‐h and 48‐h post‐UVB exposure time points, the CELLADEEP Patch group showed significant increases in R2, R5, and R7 values compared to the negative control group (*p* < 0.05). It indicates that the application of CELLADEEP Patch led to skin improvement.

In histological analysis of ex vivo human‐derived skin tissue models using optical microscopy, the CELLADEEP Patch group showed decreased epidermal thickness compared to the negative control group, with no significant structural or morphological changes noted. Additionally, the CELLADEEP Patch group showed increased collagen and elastin compared to the negative control group. Furthermore, optical microscopy observations in the CELLADEEP Patch group showed decreased melanin compared to the negative control group.

And mRNA expression analysis in the ex vivo human‐derived skin tissue model indicated that the CELLADEEP Patch group showed significant increases in COL1A1 and HAS‐3 mRNA expression levels and significant decreases in MMP‐1 and IL‐1ß mRNA expression levels compared to the negative control group (*p* < 0.05).

Altogether, the test product “CELLADEEP Patch” exhibited skin‐improving efficacy (wrinkles, inflammation, whitening, and moisturizing) in the ex vivo human‐derived skin tissue model.

## CONFLICT OF INTEREST STATEMENT

The authors declare no competing interests in this study.

## ETHICAL STATEMENT

This study has been approved by the Ethics Committee of the Korea Skin Clinical Research Center, H&Bio Corporation, Seongnam, Korea.

## Data Availability

The data that support the findings of this study are available from the corresponding author upon reasonable request.
